# Socioeconomic conditions across life related to multiple measures of the endocrine system in older adults: Longitudinal findings from a British birth cohort study

**DOI:** 10.1016/j.socscimed.2015.11.001

**Published:** 2015-12

**Authors:** David Bann, Rebecca Hardy, Rachel Cooper, Hany Lashen, Brian Keevil, Frederick C.W. Wu, Jeff M.P. Holly, Ken K. Ong, Yoav Ben-Shlomo, Diana Kuh

**Affiliations:** a MRC Unit for Lifelong Health and Ageing at UCL, London, UK; b Centre for Longitudinal Studies, UCL Institute of Education, London, UK; c Department of Human Metabolism, The University of Sheffield, Sheffield, UK; d Andrology Research Unit, School of Biomedicine, University of Manchester, UK; e School of Clinical Science, Bristol University, Bristol, UK; f MRC Epidemiology Unit, University of Cambridge, Cambridge, UK; g School of Social and Community Medicine, Bristol University, Bristol, UK

**Keywords:** Socioeconomic factors, Testosterone, Insulin-like growth factors, Thyroid, Cortisol

## Abstract

**Background:**

Little is known about how socioeconomic position (SEP) across life impacts on different axes of the endocrine system which are thought to underlie the ageing process and its adverse consequences. We examined how indicators of SEP across life related to multiple markers of the endocrine system in late midlife, and hypothesized that lower SEP across life would be associated with an adverse hormone profile across multiple axes.

**Methods:**

Data were from a British cohort study of 875 men and 905 women followed since their birth in March 1946 with circulating free testosterone and insulin-like growth factor-I (IGF-I) measured at both 53 and 60–64 years, and evening cortisol at 60–64 years. Indicators of SEP were ascertained prospectively across life—paternal occupational class at 4, highest educational attainment at 26, household occupational class at 53, and household income at 60–64 years. Associations between SEP and hormones were investigated using multiple regression and logistic regression models.

**Results:**

Lower SEP was associated with lower free testosterone among men, higher free testosterone among women, and lower IGF-I and higher evening cortisol in both sexes. For example, the mean standardised difference in IGF-I comparing the lowest with the highest educational attainment at 26 years (slope index of inequality) was −0.4 in men (95% CI -0.7 to −0.2) and −0.4 in women (−0.6 to −0.2). Associations with each hormone differed by SEP indicator used and sex, and were particularly pronounced when using a composite adverse hormone score. For example, the odds of having 1 additional adverse hormone concentration in the lowest compared with highest education level were 3.7 (95% CI: 2.1, 6.3) among men, and 1.6 (1.0, 2.7) among women (P (sex interaction) = 0.02). We found no evidence that SEP was related to apparent age-related declines in free testosterone or IGF-I.

**Conclusions:**

Lower SEP was associated with an adverse hormone profile across multiple endocrine axes. SEP differences in endocrine function may partly underlie inequalities in health and function in later life, and may reflect variations in biological rates of ageing. Further studies are required to assess the likely functional relevance of these associations.

## Highlights

• Socioeconomic position (SEP) across life was related to multiple hormone measures.
• Lower SEP was related to lower testosterone in men, higher testosterone in women.
• Lower SEP was related to lower IGF-I and higher evening cortisol in both sexes.
• SEP differences in multiple hormone axes may underlie later life health inequality.

## 1 Introduction

Socioeconomic inequalities in premature mortality, as well as physical and cognitive function are known to be substantial in the UK and many other developed nations (Hurst et al., 2013, Mackenbach et al., 2014, Schoeni et al., 2005). However, less is known about the biological mechanisms which underlie these inequalities; that is, how exposure to socioeconomic conditions in both early and adult life become embodied, leading to pathophysiological changes, impairment, disability and disease (Hertzman, 1999, Shonkoff et al., 2009).

The endocrine system, as assessed by circulating hormone concentrations in multiple axes, is influenced by environmental factors operating in both early and adult life, and has important roles in regulating both early life development and adult health and function (Ben-Shlomo et al., 2005, Cappola et al., 2007, Parekh et al., 2010). For example, low testosterone has been associated with adverse health outcomes among men, and high testosterone has been linked to adverse outcomes among women. (Araujo et al., 2011, Brand et al., 2011, Holland et al., 2011, O'Connell et al., 2011). Low IGF-I has been related to worse physical and cognitive function, and both high and low IGF-I associated with increased risk of cancer and cardiovascular mortality (Birnie et al., 2012, Burgers et al., 2011, Westwood et al., 2014). Cortisol measures (including a blunted ‘diurnal drop’) have also been associated with worse physical function (Gardner et al., 2013). The endocrine system may be especially important at older ages—changes in both its regulation and hormone production have been hypothesised to be key potential mechanisms underlying the ageing process and its adverse consequences (Chahal and Drake, 2007, Ferrucci and Studenski, 2012, Hertoghie, 2005). For example, circulating IGF-I and testosterone are thought to decline with age, while cortisol concentrations may be higher at older ages (Bann et al., 2014b, Bann et al., 2015, Chahal and Drake, 2007, Kaufman and Vermeulen, 2005). Differences in endocrine function, and in rates of age-related changes, could therefore partly underlie socioeconomic inequalities in health and function. SEP may be related to an adverse hormone profile across multiple axes, which may have additive and/or synergistic effects on physical and cognitive function in old age (Cappola et al., 2009, Hertoghie, 2005). The causal nature of hormone and health outcome relationships is not well-established and may differ by outcome given the multiple actions of hormones on different sites of the body. Not all observational studies have found expected associations between hormones such as IGF-I or cortisol and health-related outcomes (Singh-Manoux et al., 2014, Stenholm et al., 2010). While there is consistent evidence from trials in hypogonadal men that testosterone administration leads to gains in muscle and/or losses in fat mass (Isidori et al., 2005), and revitalised sexual function (Corona et al., 2014), a causal effect of endogenous testosterone on cardiovascular disease risk was not supported by a recent Mendelian randomisation study (Haring et al., 2013). In addition to potential causal roles in affecting some health outcomes, understanding the links between SEP and hormones could help to identify if some population groups may be especially vulnerable to unwarranted pharmacological hormone supplementation (eg, of testosterone among older men(Gan et al., 2013)).

Both material and psychosocial explanations for health inequalities (Bartley, 2008) could potentially be partly mediated by endocrine function, although the robustness of the links between hormones and material and psychosocial factors are uncertain. For example, some studies have found that health-impacting behaviours such as physical activity are related to sex hormones (eg, lower testosterone among women), (Rinaldi et al., 2014) while other studies have reported null associations (Sowers et al., 2001). Similarly, psychosocial factors such as stress and adverse life events have been found to relate to higher cortisol concentrations in some(Gerritsen et al., 2010) but not all studies (Ockenburg et al., 2015). Most existing studies have found that indicators of lower socioeconomic position are related to circulating hormone concentrations, including lower testosterone among men,(Zarotsky et al., 2014) higher testosterone among women (Cappola et al., 2007), lower IGF-I (Kumari et al., 2008), or higher cortisol (Castro-Diehl et al., 2014, Desantis et al., 2015, Franz et al., 2013, Kumari et al., 2010a, Miller et al., 2009). Most studies have examined cortisol, with some studies finding lower SEP was associated with lower cortisol,(Dowd et al., 2011, Groffen et al., 2015) and others finding lower SEP was associated with a less steep diurnal decline in cortisol (Desantis et al., 2015, Groffen et al., 2015, Karlamangla et al., 2013, Kumari et al., 2010a) While these separate studies suggest that SEP may be related to an adverse hormone profile across multiple endocrine axes, this requires confirmation in a single population study, particularly given null findings in some studies (Yeap et al., 2012). Existing studies have examined hormone measures at a single time point, leading to uncertainty in the relations between SEP and the age-related change in endocrine function. Most have used single indicators of SEP in adulthood (such as education or income), leading to further uncertainty as to the period/s of life in which SEP may influence the endocrine system. Of the studies which have examined SEP in childhood, relations have been found with IGF-I and cortisol, suggesting that SEP in both early and adult life may be important for subsequent endocrine axes (Franz et al., 2013, Gustafsson et al., 2010, Kumari et al., 2008, Miller et al., 2009). There may be cumulative adverse effects of low SEP across life on endocrine axes if socioeconomically patterned risk factors have independent effects on endocrine outcomes. For example, childhood SEP may capture exposure to early life stress, poor nutrition and growth, which may go on to affect endocrine function in later life independent of adult risk factors (Bann et al., 2014a, Ben-Shlomo et al., 2005, Gerritsen et al., 2010, Pearce et al., 2011).

In this study, we examined associations between several indicators of SEP (ascertained prospectively across life) with multiple components of the endocrine system, and hypothesised that lower SEP across life would be associated with an adverse hormone profile across multiple axes. We choose to examine three related endocrine pathways which we felt are likely to play an integrated role in growth, maturational development, reproduction and responsiveness to stressors. From an evolutionary perspective the ability to respond to adverse environmental circumstances would require more rapid growth, early reproductive capacity and mobilisation of metabolic resources. Both animal studies and limited human evidence suggest inter-play between the stress and reproductive axis with development being a crucial intermediary step in survival (Fitzgerald et al., 1996, Macadams et al., 1986; Robert M Sapolsky, 1985; Robert M. Sapolsky, 2005). We tested our hypotheses using data from a British birth cohort study which benefits from measures of the reproductive (testosterone), somatotrophic (insulin-like growth factor-I, IGF-I), and stress axis (cortisol). Data for testosterone and IGF-I were also obtained at two time points in midlife and early old age. We hypothesized that lower SEP across life would be associated with lower testosterone among men and higher testosterone among women and, in both sexes, with lower IGF-I, higher night time cortisol, and larger age-related declines in testosterone and IGF-I. These relations would be consistent with inequalities in health outcomes previously identified in this cohort. For example, lower SEP has been related to increased risk of premature mortality,(Kuh et al., 2002) biomarkers for cardiovascular disease risk,(Jones et al., 2015) higher fat mass (and abdominal fat distribution) (Bann et al., 2014a), and lower physical and cognitive capability (Hurst et al., 2013).

## 2 Methods

### 2.1 Study sample

The MRC National Survey of Health and Development (NSHD) is a socially stratified sample of 5362 singleton births that took place in one week of March 1946 in mainland Britain; All births from women with husbands in non-manual and agricultural employment were included, and a random selection of one in four births to females with husbands in manual employment (Wadsworth et al., 2003). The cohort has been followed-up a total of 24 times across life, with hormone data derived from blood samples at 53 and 60–64 years. At 60–64 years, 2856 eligible study members were invited for an assessment at one of six clinical research facilities (CRF) or a home visit. Invitations were not sent to those who had died (n = 778), who were living abroad (n = 570), had previously withdrawn from the study (n = 594) or who had been lost to follow-up (n = 564). Of those invited, 2229 were assessed: 1690 attended a CRF and the remaining 539 were seen at home (Kuh et al., 2011). Due to an increased likelihood of premature mortality (Kuh et al., 2002) and loss to follow-up (Stafford et al., 2013a), those of lower SEP were less likely to participate in this wave. The study received Multi-Centre Research Ethics Committee approval and informed consent was provided by participants.

### 2.2 Blood and saliva sampling, and hormone measurement

The standardized blood sampling and measurement of testosterone (Bann et al., 2015), IGF(Bann et al., 2014b) and cortisol (Kirschbaum and Hellhammer, 1989, Stafford et al., 2013b) in the NSHD have been described in detail elsewhere and are briefly outlined below.

At 53 years, non-fasting venous blood samples were taken in EDTA tubes during home visits by research nurses in the morning (27%), afternoon (30%), or evening (43%). These were posted overnight to a laboratory where plasma was extracted and frozen at −80 °C. At 60–64 years, overnight fasting venous blood samples were obtained in citrate tubes, mostly in the morning (93% between 7.30am and 10am). These were then taken immediately to a laboratory in each CRF or posted overnight to a CRF (home visit) and plasma extracted before being frozen at −80°C—plasma samples were transported between laboratories on dry ice to prevent thawing. Plasma samples for both 53 and 60–64 years were assayed during the same period.

IGF-1, IGF-II, and IGFBP-3 were measured from plasma samples at 53 and 60–64 years using radioimmunoassay. Intra-assay COV were 3.4% (IGF-I), 2.8% (IGF-II), and 3.9% (IGFBP-3); inter-assay COV were 13.7% (IGF-I), 7.4% (IGF-II), and 11.7% (IGFBP-3). The remaining plasma samples from 53 to 60–64 years were then assayed for testosterone, using liquid chromatography–mass spectrometry, and its binding protein, sex-hormone binding globulin (SHBG) using immunoassay (inter-assay COVs both <5.4%). Since sizable proportions of circulating testosterone are chemically bound to SHBG, and not able to affect target tissue, free testosterone was calculated using the formula described by Vermeulen et al (Vermeulen et al., 1999). Because testosterone varies diurnally—concentrations are substantially higher in the morning than the afternoon or evening (P < 0.05)—we estimated morning testosterone concentrations at 53 years for all participants to aid comparability with 60–64 year samples (Bann et al., 2015). This was achieved by constructing sex-specific regression models with testosterone at 53 years as the outcome and time of blood collection as a categorical exposure; the model residuals were then added to mean morning testosterone concentration for those who gave blood samples in the afternoon and evening. Unlike testosterone, IGF-I concentrations did not differ according to the timing of the blood sample (P = 0.85).

At 60–64 years, cortisol was measured using radioimmunoassay of saliva samples(Kirschbaum and Hellhammer, 1989) obtained in the evening (9–9.30pm), and at usual waking and 30 min after the following day. Participants were asked not to drink 30 min before providing the sample and not smoke, eat, or brush their teeth until after all samples were provided. Intra- and inter assay coefficients of variation were below 10%. Due to protocol changes, cortisol was not measured in the first clinical assessment (N = 230). Participants who reported taking steroids at 60–64 years were excluded from analyses (n = 15), as were those with outlying values (<or > 4 SDs from the mean, N = 23).

### 2.3 Socioeconomic position across life

Indicators were selected a-priori to capture SEP across life. Paternal occupational class at 4 years was used as an indicator of childhood SEP (the Registrar General's classification (RGSC) consisting of 6 standard categories—I professional, II intermediate, III skilled non-manual, III skilled manual, IV semi-skilled, and V unskilled. Highest educational level achieved by 26 years was classified using the Burnham scale(Department of Education and Science (1972)): 1) no qualifications, 2) CSE, clerical course or equivalent, 3) O level or equivalent (age 16 years), 4) A level or equivalent (age 18 years), and 5) Degree or higher (Department of Education and Science (1972)) Two other measures of adult SEP were used: highest household occupational class at 53 years (RGSC of the participant or their partner) and, after most participants had retired (∼61%), self-reported household income at 60–64 years were used to ascertain contemporaneous SEP (post-tax from all sources in 13 bands from per year: £≤6000 to ≥30000).

### 2.4 Analytical strategy

We conducted main analyses using a single circulating hormones measure from each axis which we expected to be most closely related to health and functional outcomes in older age: free testosterone, IGF-I, and evening cortisol; associations with total testosterone, SHBG, IGF-II and IGFBP3, and other cortisol measures (morning concentration and diurnal drop) are presented in supplementary tables.

We examined crude estimates of association by calculating average hormone concentrations at 60–64 years by SEP categories, and by examining associations between each SEP indicator and each hormone using linear regression. Medians were used due to the right-skew of some hormone measures. To facilitate comparisons of the associations across different SEP indicators, slope indices of inequality (SII) were then used,(Mackenbach and Kunst, 1997) which account for differences in the distribution of participants across categories of different SEP indicators. Each indicator was converted into sex-specific ridit scores; these range from 0 to 1, with each SEP category given a value equivalent to the proportion of the population with higher SEP than the midpoint of that category. These ridit scores were then used as exposures in linear regression models—the coefficients represent the absolute difference in outcome when comparing those with the hypothetically lowest (1) with hypothetically highest SEP (0) (Mackenbach and Kunst, 1997). To aid the interpretation of effect sizes, all hormone measures were converted into standard deviation scores (z-scores); measures which were right-skewed (testosterone in women, and cortisol in both sexes) were first log-transformed. To examine whether SEP across life was cumulatively associated with hormones, we examined associations with a lifetime SEP score, created by summing all SEP ridit scores and dividing by 4 (so that the scales were equivalent with models using single SEP indicators). These models were examined and compared with models examining single SEP indicators with respect to statistical significance (using likelihood ratio test comparisons with the null model) and model fit (using Akaike Information Criterion (AIC)). Lower AIC values indicate an improved model fit, yet there are no well-established cut-points for determining the extent to which an improved model fit is large, moderate, or small. As both low and high hormone concentrations could indicate impaired endocrine regulation, we also examined whether SEP was associated with both high and low hormone concentrations; SEP indicators were cross-tabulated with hormones categorized as the lowest 12.5%, middle 75%, and highest 12.5%, and separate logistic regression models were conducted to test associations between SEP with risk of both low and high hormone concentrations.

To examine whether SEP was related to an adverse hormone profile across multiple axes, we created a composite hormone score (Cappola et al., 2009). This was calculated by converting hormone measures into binary scores; a score of 1 was given if participants were in the lowest quartile for IGF-I or the highest quartile for evening cortisol. Men in the lowest quartile of free testosterone were also given a score of 1, while women in the highest quartile were given a score of 1 given evidence for sex-divergent effects of testosterone (Brand et al., 2011). These scores, ranging from 0 (most optimal hormone profile) to 3 (most adverse profile), were then cross-tabulated with SEP indicators, and used as an outcome in ordered logistic regression models. The proportional odds assumption was tested using a likelihood ratio test to compare constrained and unconstrained models.

For the hormones measured repeatedly (IGF and testosterone), we also examined whether SEP up to 53 years (and a lifetime SEP score up to 53 years) was associated with hormone concentrations at 53 years and with calculated changes in hormone concentrations between 53 and 60–64 years.

Because the determinants of hormone concentration may differ by sex,(Parekh et al., 2010) we conducted all analyses separately in men and women, and formally tested sex by SEP interaction terms. Given evidence for sex differences in some associations with testosterone and IGF-I (P < 0.05), we stratified main analyses examining these hormones by sex. Deviation from linearity was also assessed using likelihood ratio tests. Sample sizes (male/female) for each hormone at 60–64 years were as follows: 865/888 for testosterone, 875/905 for IGF-I, and 835/943 for cortisol. Analyses were restricted to participants with valid data for the SEP indicator/s used and each hormone measure. Missing outcome data was attributable to a number of reasons including insufficient plasma samples (IGF and testosterone data were measured after other biochemical assays) and procedural and technical issues. In addition, cortisol was not measured in the first clinical assessment facility (N = 230), and some participants did not provide a blood sample (n = 86). The statistical package Stata (Stata Corporation, Texas, USA) was used for all analyses.

### 2.5 Additional and sensitivity analyses

To examine whether the categorization of hormones affected associations between SEP and the composite hormone score, analyses were repeated using different cut-points to categorize the top or bottom 15% of each hormone, instead of 25%. To examine the extent to which the use of estimated morning testosterone levels (age 53 years) for participants who gave samples in the afternoon or evening impacted on findings, additional analyses were conducted using raw testosterone values in which the time of blood sampling was not accounted for. Finally, to examine whether missing data affected findings, we conducted additional analyses using i) multiple imputation and ii) full information maximum likelihood estimation.

## 3 Results

Men had higher free testosterone and IGF-I concentrations at 60–64 years than women, while there was no evidence for a sex difference in evening cortisol (P = 0.7; Table 1). Mean free testosterone and IGF-I concentrations were lower at 60–64 than at 53 years in both men and women, as previously described (Bann et al., 2014b, Bann et al., 2015). Among both sexes, there was variation in the number of adverse hormone concentrations observed (in men: 0 = 45.9% 1 = 37.4%, 2/3 = 16.7%; women: 0 = 43.7%, 1 = 41.0%, 2/3 = 15.3%). Those with missing hormone data differed with respect to their socioeconomic profile—they were more likely to have been of lower SEP (P < 0.01 for all SEP indicators and outcomes). Correlations between the different hormone outcomes were weak—cortisol was not associated with IGF-I or free testosterone (r < 0.1 in both sexes), and IGF-I and free testosterone were weakly positively correlated (r = 0.1 in men and 0.2 in women).

### 3.1 Testosterone

Among men, lower education and lower income were associated with lower testosterone at 60–64 years, while childhood SEP and occupational class were not associated (Table 1, Table 2). For example, results suggested a −0.37 (95% CI: -0.61, -0.12) standard deviation lower free testosterone concentration in those with lowest compared with highest educational attainment. In contrast, among women lower childhood SEP was associated with higher testosterone (p for sex interaction = 0.01), while other indicators were not associated. Models including single indicators of SEP (income in men and childhood SEP in women) provided a better model fit (lower AIC) than a model using the lifetime SEP score, suggesting little evidence for a cumulative association.

### 3.2 IGF-I

Lower education was associated with lower IGF-I at 60–64 years among men (Table 1, Table 2), while other indicators and the lifetime SEP score were not significantly associated. Among women, lower education and income were associated with lower IGF-I, and a model using a lifetime SEP score provided the best model fit (marginally), suggesting a cumulative association between lower SEP across life and lower IGF-I. For example, results suggested a −0.59 (−0.95, -0.22) standard deviation lower IGF-I amongst those with lowest compared with highest socioeconomic position across life.

### 3.3 Cortisol

A lower lifetime SEP score was associated with higher evening cortisol at 60–64 years (β = 0.32 (95% CI: 0.07–0.56; AIC = 4245.7)), but this model had a slightly higher AIC than the model solely examining education (β = 0.22 (0.05–0.39); AIC = 4244.6). However, in both relative and absolute terms, the AIC was only marginally smaller. Other indicators of SEP were more weakly associated with higher evening cortisol, and 95% CIs overlapped with the null.

### 3.4 Composite adverse hormone score across multiple axes

Cross-tabulations and logistic regression results indicated no evidence that SEP was associated with either low or high hormone concentrations (data available on request).

**Fig. 1:**
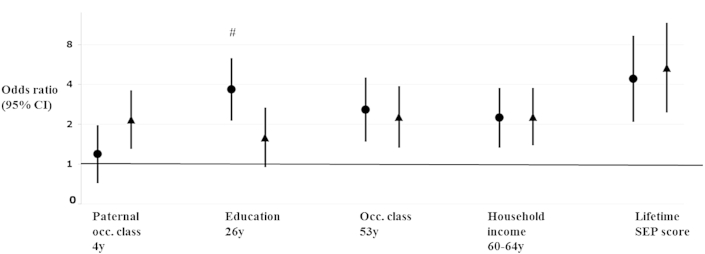
Socioeconomic position in relation to a composite hormone score at 60–64 years—odds ratios (95% CI) of being in a 1-unit higher (adverse) hormone profile score in the lowest compared with highest socioeconomic position (relative index of inequality). Note: men shown in circles, women in triangles. Hormone profile scores were calculated by adding together the number of adverse hormone concentrations recorded at 60–64 years (0, 1, 2–3). A score of 1 was given if participants were in the lowest quartile for either IGF-I, or free testosterone concentration (among men only—among women those in the highest quartile were given a score of 1), or the highest quartile of evening cortisol concentration. Odds ratios were calculated using ordered logistic regression. Sample sizes were as follows: paternal occupational class (648 men, 682 women), education (638/690), occupational class (670/703), household income (656/689), and lifetime SEP score (601/609); #p-value for sex interaction term <0.05.

Associations between SEP and the composite adverse hormone score followed the same pattern as associations with its individual components (Fig. 1). Lower SEP in childhood (more strongly in women than men (P(sex interaction) = 0.12)), lower education, lower occupational class, and lower household income were associated with higher adverse hormone scores. For example, in the lowest compared with highest education, the odds of having 1 additional adverse hormone concentration was 3.67 (95% CI: 2.13, 6.31) among men, and 1.59 (0.95, 2.67) among women (P (sex interaction) = 0.02). There was little evidence for deviation from the proportional odds assumption (p-values from likelihood ratio tests all >0.15), or for deviation from linearity in the above models.

### 3.5 SEP across life and estimated mid-life decline in IGF-I and testosterone

SEP was not significantly related to testosterone at 53 years among men, while among women only low childhood SEP was related to higher testosterone at 53 years (β = 0.61, 95 CI: 0.31, 0.92); Table 3. However, SEP was not significantly related to change in testosterone in either sex between 53 and 60–64 years. Among men, lower education and occupational class were more weakly associated with lower IGF-I at 53 years, compared with associations with IGF-I at 60–64 years. Among women, only lower occupational class was significantly associated with lower IGF-I at 53 years. There was little evidence that SEP was associated with change in IGF-I between 53 and 60–64 years in either men or women.

### 3.6 Additional and sensitivity analyses

Associations between SEP and total testosterone were similar to those with estimated free testosterone (supplementary table 1). Lower SEP was weakly and not-significantly associated with lower SHBG and lower IGF-II, and lower income was associated with lower IGFBP3 in both sexes; indicators of SEP were generally not associated with morning cortisol or diurnal drop (supplementary table 1). Conclusions did not differ when categorizing adverse hormone concentrations as being in the 15% (rather than 25%) highest or lowest hormone concentrations, when not adjusting for time of blood collection time at 53 years, nor when either using multiple imputation or full information maximum likelihood estimation to account for missing data on SEP indicators.

## 4 Discussion

### 4.1 Main findings

In a nationally representative British birth cohort study assessed in late midlife, we found evidence that lower levels of some indicators of SEP were associated with an adverse hormone profile across multiple axes: with lower testosterone in men and higher testosterone in women, and with lower IGF-I and higher evening cortisol in both sexes. However, associations found with each hormone differed by SEP indicator used and sex; among women our data suggested a cumulative association between lower SEP across life and lower IGF-I, while there was no strong evidence for accumulation in associations with testosterone or cortisol. Associations between SEP and hormones were particularly pronounced when using a composite adverse hormone score.

### 4.2 Comparison with previous studies

Findings from this study add to the previous evidence relating SEP to single hormone axes. For example, separate analyses have shown that lower SEP is associated with lower testosterone among men,(Zarotsky et al., 2014) higher testosterone among women,(Cappola et al., 2007) lower IGF-I,(Kumari et al., 2008) or higher cortisol concentrations (Castro-Diehl et al., 2014, Desantis et al., 2015, Franz et al., 2013, Kumari et al., 2010a, Miller et al., 2009). We extend these findings by: better characterising the relations between SEP and hormones in a well-characterised representative birth cohort study; examining multiple hormones from different axes; using repeat measures of IGF-I and testosterone; and by using multiple indicators of SEP ascertained prospectively across life which enabled us to test for cumulative associations. Our findings add to the understanding of how SEP relates to biological processes which are thought to partly underlie the ageing process and its adverse consequences. These findings are broadly consistent with previous evidence from this cohort which has identified socioeconomic inequalities in health-related outcomes which may be partly explained via an influence on the endocrine system (Bann et al., 2014a, Hurst et al., 2013, Jones et al., 2015, Kuh et al., 2002).

### 4.3 Explanation of findings

Associations between SEP and multiple hormones indicate the socioeconomic patterning of the lifetime determinants of the endocrine systems. The hormones investigated are known to be tightly regulated by a series of homeostatic mechanisms (feedback loops)—the presence of associations suggests that socioeconomically patterned risk factors may have affected the homeostatic set-points by which typical hormone concentrations are maintained, and/or affected the regulation of the homeostatic mechanisms. These risk factors may be both shared across different axes and/or affect only single hormone outcomes. For example, lower SEP is related to higher fat mass levels,(Bann et al., 2014a) which in turn may lead to lower IGF-I,(Bann et al., 2014b) lower testosterone in men and higher testosterone in women (Bann et al., 2015). In contrast, higher fat mass has been found to be non-linearly associated with cortisol (Kumari et al., 2010b). Other factors which may lie on the pathway between SEP and hormones may be health-impacting behaviours such as physical activity, diet, and smoking, exposure to stress and adverse events, age at menopause,(Hardy and Kuh, 2005) and incidence of clinically defined and sub-clinical ill health. Early life SEP may be related to hormones due to the persistence of socioeconomic conditions across life since low childhood SEP typically limits opportunities with respect to educational attainment, occupation, income and wealth. Childhood SEP may also capture the influence of childhood exposures which have persisting effects on hormones independent of subsequent SEP. For example, childhood milk intake has been related to adult IGF-I concentrations in old age,(Ben-Shlomo et al., 2005) and exposure to stress in both early and adult life has been related to adult cortisol concentrations (Gerritsen et al., 2010). Delineating the relative contributions of these different pathways is likely to be challenging given the potential for bi-directionality in associations between many risk factors and hormones (eg, between lower testosterone and ill health). Although we hypothesized that the principal direction of association was from SEP to hormones, it is possible that associations may be explained by reverse causality, or be bi-directional in nature. For example, systemic disease may lead to lower testosterone in men,(Karagiannis and Harsoulis, 2005) which could in turn limit future occupational and earning opportunities, and thereby confound associations between SEP with subsequent hormone outcomes. Reverse or bi-directionality is however less likely to explain the prospective associations found between childhood SEP and subsequent hormones, as the childhood SEP measures used (ascertained at 4 years) preceded the hormone measures by up to 60 years. Confounding by genetic factors which affect both circulating hormones and socioeconomic outcomes may also have a role in explaining our findings, although hormones (and socioeconomic factors) are known to be affected by environmental factors (Ben-Shlomo et al., 2005, Cappola et al., 2007, Parekh et al., 2010).

Associations between SEP indicators and testosterone among men were stronger (and more significant) at 60–64 years than at 53 years. This may suggest that the socioeconomic patterning of endocrine function determinants may become especially pronounced at older ages. However, associations of SEP with changes in testosterone and IGF-I were not significant. These may reflect true null findings or lack of power as the age-related decline will be partially masked by measurement error and regression to the mean, particularly when only 2 time points are available (Singer and Willett, 2003). Further studies are required with more frequently repeated samples to better characterise these dynamic relationships in later decades. Differences in the strength and significance of association between each SEP indicator and hormones may be explained by differences in the extent to which each SEP indicator relates to the above intermediary risk factors—each captures information during particular periods of life, and in different aspects of the socioeconomic environment. For example, among men educational attainment was more strongly associated with fat mass at 60–64 years in this cohort than paternal occupational class,(Bann et al., 2014a) potentially explaining its stronger association with testosterone and IGF-I. Among men, lower income was most strongly related to lower testosterone, while among women only lower childhood SEP was related to higher testosterone. These findings suggest that aspects of contemporaneous SEP in older age and/or factors related to adulthood material circumstances may have especially impacted on testosterone concentrations in men, whilst in women developmental influences may have impacted on long term testosterone concentrations. Contrary to expectation, we did not find strong evidence that childhood SEP was associated with other outcomes. This could indicate that contemporaneous adult socioeconomic circumstances are stronger determinants of the hormone concentrations investigated, potentially due to the combined influence of material, behaviours, and/or psychosocial pathways. Lower education was most strongly related to higher evening cortisol, suggesting that factors especially related to education may have impacted on cortisol concentrations. Alternatively, education may have simply been the most relevant marker of SEP across life, as it reflects both childhood experiences and subsequent employment, income, and wealth opportunities (Galobardes et al., 2006). Indeed, education has been considered a ‘fundamental cause’ of health inequality, since the links between education and health have persisted despite changes over time in the benefits which a higher education attainment provides (Montez and Friedman, 2015). Taken together, our findings suggest that studies using single indicators of SEP may provide an incomplete understanding of how SEP impacts on endocrine function. Although this warrant future consideration, caution is needed when interpreting associations with multiple SEP indicators, as some results could reflect chance findings as multiple statistical tests have been carried out; the interpretation of results may be more appropriately focused on the overall patterns of estimated associations (and corresponding confidence intervals) rather than the significance level of each specific association examined.

### 4.4 Strengths and limitations

Strengths of this study include the availability of prospectively ascertained SEP indicators obtained across life and multiple hormone measures, some of which were repeated. While multiple different SEP indicators were used which have been found to relate to health-related outcomes in this cohort, other research may be required to more comprehensively examine which dimensions of SEP are most closely related to endocrine functioning (eg, with respect to education, occupational class, income, or wealth). As in all longitudinal studies, loss to follow-up occurred which reduced statistical power and may have introduced bias. Specifically, study members with lower SEP and worse functional health were less likely to participate at 60–64 years, through death or attrition (Cooper et al., 2014; Stafford et al., 2013a). If those lost to follow-up also had especially unfavourable hormone profiles, this pattern of missing data may have weakened the observed associations between SEP and hormones; in other cases, missing data could have led to strengthening of the observed relationships. Sample sizes for repeat testosterone measures were limited by the numbers of remaining stored serum samples. However, the loss in sample size and power may have been partly offset by the comparatively higher accuracy and precision of the mass-spectroscopy assays used—unlike immunoassay, mass spectroscopy has sufficient sensitivity to accurately quantify the low testosterone levels among women (Gallagher et al., 2007, Thienpont et al., 2008).

Because of day-to-day and week-to-week variability in hormone concentrations, the single blood (and one-day saliva) sample measures obtained at each age imperfectly captured long-term concentrations. Assuming that this source of error was random, it would have led to less precise estimates of association between SEP and hormones, particularly where COV were higher (eg, for IGF-I). In addition, the limited number of cortisol measures used are likely to have imperfectly captured the true diurnal variability in cortisol which may be more closely related to SEP and health outcomes. However, other studies have found that cortisol measures in the NSHD relate to physical function and social isolation, suggesting that the measures do have moderate-good convergent validity (Gardner et al., 2013, Stafford et al., 2013b). Methodological differences in blood sampling at 50 and 60–64 years (eg, non-fasting samples at 53 years obtained across the day) may have impacted on the results examining change in hormones, although we found little evidence that accounting for blood sample time affected findings. Although we investigated multiple hormones in this study, only IGF and testosterone were measured at two ages, as such we were unable to examine how SEP related to change in cortisol during age. In order to fully understand how SEP relates to change in hormone measures during the ageing process, more repeated measurements are clearly required, spanning early and later life. Aside from the measures included in this study, other hormones may also be important for health and function in older age, and may therefore warrant future investigation. For example, estrogen was not measured in the NSHD, precluding its investigation here despite its potential importance for health among women (Corbelli et al., 2015, Herrera and Mather, 2015). Measures of thyroid function are also likely to warrant future investigation: lower SEP has been related to increased hyperthyroidism risk,(Wilson et al., 2006) which may in turn predict adverse health, although the prevalence of hyperthyroidism was low in the NSHD at 60–64 years (1.7%) (Pierce et al., 2012). We used a composite score to describe the number of adverse hormone concentrations—similar composite scores have been shown to be more strongly related to frailty risk than single hormone measures,(Cappola et al., 2009, Hertoghie, 2005) but they are clearly simplifications of a dynamic, interactive endocrine system, and their construction is partly dependent on the hormones measured in each study. If associations between exposures and hormone outcomes are independent of other hormones, it would be expected that associations with a composite hormone outcome would be stronger. However, it should be noted that there is no accepted standard method to either define or measure multiple adverse hormone concentrations, nor conceptual phenomena such as dysregulation (Ferrucci and Studenski, 2012). Further developments may enable more detailed characterisation of endocrine function across multiple axes, including the use of dynamic measures which arguably have more relevance to biological function than single measures of circulating concentrations.

### 4.5 Implications

Findings from this study suggest that those of lower SEP may be at higher risk of having adverse hormone concentrations across multiple axes in late midlife. These associations may in turn partly underlie socioeconomic inequalities in a range of health and functional outcomes in older age as the endocrine system has been hypothesised as being a key modulator of the ageing process (Chahal and Drake, 2007, Ferrucci and Studenski, 2012, Hertoghie, 2005). Future studies are needed to empirically test these mediating pathways. While the identification of underlying biological mechanisms may help us to better understand the effects of SEP across life and help strengthen causal inferences, mechanistic understanding is not a prerequisite for successful intervention—there is increasing evidence that interventions targeting the up-stream socioeconomic factors are more efficacious in reducing socioeconomic inequalities in health than those which target intermediary factors (Lorenc et al., 2013).

The relative importance of these and other hormones in human ageing is currently uncertain. While IGF-I and testosterone have been observed to decline with age, and be related to health and functional outcomes, important heterogeneity exists, with some studies not finding expected associations between testosterone, IGF-I or cortisol(Singh-Manoux et al., 2014, Stenholm et al., 2010) and health-related outcomes. This, in addition to potential publication bias which could favour significant relations between hormones and health-related outcomes in observational studies, suggests that future experimental and observational studies are needed to help delineate the relative importance of hormonal systems for the ageing process and its consequences.

### 4.6 Conclusions

In a British birth cohort study, lower SEP was associated with an adverse hormone profile in late midlife age across multiple axes (lower testosterone among men, higher testosterone among women, and with lower IGF-I and higher evening cortisol in both sexes). Associations with individual hormones differed by SEP indicator and sex. Further studies are required to better characterize how SEP relates to age-related changes in endocrine function, and to examine the functional relevance of associations found.

## Conflict of interest

Nothing to declare.

## Figures and Tables

**Table 1:** Hormone concentrations at 60–64 years by indicators of socioeconomic position across life.

	N (M/F)	Free testosterone (pmol/L) Median (IQR)		IGF-I (ng/ml) Median (IQR)		Cortisol (evening; nmol/L) Median (IQR)
Men	Women	Men	Women	Both sexes
Total sample	875/905	235.0 (87.4)	7.4 (5.3)	179.0 (74.8)	158.0 (75.2)	2.41 (2.13)
*P* (*t*-*test*)			<0.001		<0.001	
Paternal occ class (4y)						
I professional	65/64	242.1 (98.9)	7.1 (4.8)	173.0 (48.6)	144.2 (59.7)	2.18 (2.15)
II intermediate	155/164	234.2 (72.8)	7.3 (5.0)	179.0 (77.1)	171.4 (74.3)	2.45 (2.17)
III skilled (Non-Manual)	167/182	235.3 (73.2)	7.1 (5.0)	185.7 (77.1)	159.4 (69.5)	2.46 (2.17)
III skilled (Manual)	242/244	233.1 (96.7)	7.2 (5.4)	170.4 (80.2)	158.6 (82.0)	2.54 (2.08)
IV partly skilled	163/157	230.2 (86.8)	8.2 (5.6)	183.9 (74.6)	152.0 (72.3)	2.34 (2.11)
V unskilled	44/47	251.2 (95.2)	8.2 (6.3)	195.0 (85.2)	153.4 (84.3)	2.54 (1.95)
*P* (trend)		0.37	<0.01	0.18	0.08	0.08
Own education (26y)						
Degree or higher	155/55	246.9 (79.1)	6.6 (5.9)	180.3 (66.2)	165.2 (90.1)	2.28 (2.42)
GCE A level	256/256	234.4 (88.8)	7.5 (4.7)	185.6 (72.7)	169.3 (82.8)	2.31 (1.98)
GCE O level	125/229	236.1 (94.5)	7.3 (5.0)	178.0 (74.8)	153.6 (68.4)	2.53 (2.39)
Sub GCE O-level	46/84	234.2 (63.2)	8.6 (5.6)	186.0 (86.5)	149.9 (84.7)	2.49 (2.43)
No qualifications	245/238	230.2 (94.5)	7.7 (6.0)	165.4 (77.4)	156.3 (70.9)	2.48 (2.10)
*P* (*trend*)		<0.01	0.91	<0.01	<0.01#	0.02
Occupational class (53y)						
I professional	137/91	240.1 (67.5)	6.9 (5.0)	186.0 (58.0)	155.2 (57.7)	2.35 (2.21)
II intermediate	425/444	235.0 (92.8)	7.1 (5.2)	175.0 (73.1)	162.0 (76.0)	2.37 (1.99)
III skilled (Non-Manual)	164/211	225.3 (78.9)	8.0 (6.0)	189.3 (73.3)	159.3 (82.3)	2.46 (2.27)
III skilled (Manual)	104/74	243.6 (89.1)	8.4 (5.6)	159.7 (88.0)	146.3 (85.2)	2.44 (2.17)
IV partly skilled	29/48	235.7 (95.0)	8.0 (4.5)	144.7 (84.4)	145.1 (65.7)	2.73 (2.79)
V unskilled	8/10	250.1 (98.8)	6.4 (3.6)	183.9 (66.0)	128.7 (67.4)	3.35 (3.87)
*P* (*trend*)		0.56	0.30	0.09	0.02	<0.01
Household income (60–64y)						
1 (highest)	322/238	247.5 (89.5)	7.2 (4.8)	182.8 (72.8)	163.4 (79.5)	2.36 (2.14)
2	209/212	233.9 (73.4)	7.5 (5.6)	177.6 (77.4)	170.9 (71.7)	2.37 (1.93)
3	197/242	231.1 (98.7)	7.4 (5.6)	177.0 (81.2)	156.7 (77.0)	2.40 (2.06)
4 (lowest)	102/159	222.5 (82.7)	7.5 (5.3)	175.5 (75.6)	146.0 (68.1)	2.53 (2.59)
*P* (*trend*)		<0.001	0.42	0.21	<0.01	0.06#

Sample sizes presented for IGF-I, and differ for testosterone (865/888) and cortisol (835/943), and by SEP indicator used; #p(heterogeneity) shown due to non-linearity (P < 0.05); log-transformed cortisol values used to calculated P-values due to right-skew; income displayed in 4 categories here to aid interpretation.

**Table 2:** Estimated standard deviation differences in hormone concentrations (95% CI) at 60–64 years between the hypothetical lowest and highest socioeconomic position (slope index of inequality).

	Free testosterone (752 men, 745 women)	P	AIC	IGF-I (760 men, 758 women)	P	AIC	Cortisol (evening) (736 men, 780 women)	P	AIC
**Men**							**Both sexes**		
Paternal occ. class (4y)	−0.10 (−0.34, 0.14)	0.40#	2102.7	0.14 (−0.09, 0.38)	0.23#	2130.8	0.10 (−0.07, 0.27)	0.24	4250.6
Own education (26y)	−0.37 (−0.61, −0.12)	<0.01#	2099.1	−0.40 (−0.65, −0.16)	<0.01	2124.6	0.22 (0.05, 0.39)	0.01	4244.6
Occupational class (53y)	−0.11 (−0.36, 0.15)	0.41	2103.8	−0.23 (−0.49, 0.02)	0.07	2129.6	0.17 (−0.01, 0.34)	0.06	4249.1
Household income (60–64y)	−0.42 (−0.66, −0.19)	<0.01#	2092.7	−0.17 (−0.40, 0.07)	0.17	2131.5	0.15 (−0.01, 0.32)	0.07	4249.1
*Lifetime SEP score*	−0.45(−0.79, −0.11)	0.01#	2097.6	−0.27(−0.62, 0.08)	0.13	2130.9	0.32(0.07, 0.56)	0.01	4245.7
**Women**									
Paternal occ. class (4y)	0.29 (0.08, 0.50)	<0.01	1944.2	−0.22 (−0.46, 0.02)	0.07	2162.2			
Own education (26y)	0.14 (−0.08, 0.35)	0.21	1948.9	−0.40 (−0.64, −0.15)	<0.01	2154.6			
Occupational class (53y)	0.15 (−0.07, 0.38)	0.18	1947.2	−0.25 (−0.50, 0.01)	0.06	2160.5			
Household income (60–64y)	0.08 (−0.-13, 0.29)	0.45	1949.2	−0.32 (−0.55, −0.09)	<0.01	2156.9			
*Lifetime SEP score*	0.38 (0.05, 0.71)	0.02	1945.1	−0.59(−0.95, −0.22)	<0.01	2154.1			

#P-value for sex interaction term <0.05; sexes combined for cortisol due to a lack of evidence for sex interaction; occupational class was that of the highest in the household and derived using the Registrar General's classification; Analyses using lifetime SEP score (and all AIC calculations) were restricted to those with valid data for all SEP indicators and each hormone.

**Table 3:** Estimated standard deviation differences in free testosterone and insulin like growth factor-I (IGF-I) concentrations at 53, 60–64 years, and change between these ages, between the hypothetical lowest and highest socioeconomic position (slope index of inequality).

	N	Testosterone, 53y	P	Testosterone, 60–64y	P	Δ Testosterone	P
**Men**							
Paternal occ. class (4y)	417	−0.03(−0.28, 0.23)	0.83	−0.21(−0.46, 0.04)	0.10	−0.18(−0.52, 0.16)	0.30
Own education (26y)	415	−0.13(−0.39, 0.13)	0.33	−0.46(−0.71, −0.20)	<0.001	−0.29(−0.64, 0.06)	0.10
Occupational class (53y)	436	−0.18(−0.44, 0.09)	0.20	−0.34(−0.60, −0.08)	0.01	−0.13(−0.49, 0.23)	0.48
*Lifetime SEP score*	400	−0.20(−0.54, 0.14)	0.25	−0.49(−0.82, −0.15)	<0.01	−0.23(−0.68, 0.22)	0.32
**Women**							
Paternal occ. class (4y)	526	0.61(0.31, 0.92)	<0.001	0.33(0.05, 0.61)	0.02	−0.11(−0.29, 0.07)	0.23
Own education (26y)	528	0.08(−0.23, 0.39)	0.63	0.09(−0.19, 0.37)	0.54	0.05(−0.13, 0.24)	0.56
Occupational class (53y)	538	−0.09(−0.42, 0.23)	0.58	0.17(−0.13, 0.47)	0.27	0.11(−0.08, 0.30)	0.25
*Lifetime SEP score*	499	0.41(−0.02, 0.83)	0.06	0.34(−0.06, 0.73)	0.09	−0.01(−0.25, 0.24)	0.96

	N	IGF-I, 53y	P	IGF-I, 60–64y	P	Δ IGF-I	P

**Men**							
Paternal occ. class (4y)	719	0.08(−0.16, 0.32)	0.52	0.12(−0.14, 0.37)	0.37	0.02(−0.24, 0.28)	0.88
Own education (26y)	713	−0.25(−0.50, 0.00)	0.05	−0.35(−0.61, −0.08)	0.01	−0.05(−0.31, 0.22)	0.73
Occupational class (53y)	746	−0.24(−0.50, 0.02)	0.07	−0.30(−0.57, −0.02)	0.03	−0.01(−0.29, 0.27)	0.94
*Lifetime SEP score*	692	−0.25(−0.58, 0.08)	0.14	−0.26(−0.61, 0.09)	0.14	0.04(−0.31, 0.38)	0.84
**Women**							
Paternal occ. class (4y)	760	−0.21(−0.47, 0.05)	0.12	−0.28(−0.53, −0.03)	0.03	−0.04(−0.29, 0.21)	0.77
Own education (26y)	767	−0.16(−0.43, 0.10)	0.23	−0.43(−0.68, −0.17)	<0.01	−0.20(−0.46, 0.06)	0.13
Occupational class (53y)	783	−0.37(−0.64, −0.10)	<0.01	−0.27(−0.54, 0.00)	0.05	0.13(−0.14, 0.40)	0.35
*Lifetime SEP score*	719	−0.37(−0.74, 0.00)	0.05	−0.56(−0.91, −0.20)	<0.01	−0.11(−0.47, 0.25)	0.54

Δ calculated as 60–64y minus 53y hormone concentration; occupational class was that of the highest in the household and derived using the Registrar General's classification.
