# Supplementary material for: Socioeconomic conditions across life related to multiple measures of the endocrine system in older adults: Longitudinal findings from a British birth cohort study
Source: Soc Sci Med. 2015 Dec;147:190–9. doi: 10.1016/j.socscimed.2015.11.001 (PMC4686046; doi:10.1016/j.socscimed.2015.11.001)
Supplement: Supplementary file 1 [file mmc1.doc]

Supplementary Table 1. Mean standard deviation differences in hormone concentrations (95% CI) at age 60–64 years between the hypothetical lowest and highest socioeconomic position (relative index of inequality).

|  | Total testosterone | P | SHBG | P | IGF-II | P |
| --- | --- | --- | --- | --- | --- | --- |
| **Men** |  |  |  |  |  |  |
| Paternal occ. class (4y) | -0.15 (-0.39, 0.9) | 0.22# | -0.05 (-0.28, 0.18) | 0.70 | -0.14 (-0.38, 0.09) | 0.23 |
| Own education (26y) | -0.40 (-0.65, -0.15) | <0.001# | -0.18 (-0.42, 0.05) | 0.12 | -0.26 (-0.50, -0.02) | 0.04 |
| Occupational class (53y) | -0.18 (-0.44, 0.08) | 0.17 | -0.09 (-0.33, 0.16) | 0.49 | -0.12 (-0.37, 0.14) | 0.36 |
| Household income (60-64y) | -0.36 (-0.60, -0.12) | <0.01# | -0.12 (-0.35, 0.11) | 0.30 | -0.14 (-0.37, 0.10) | 0.25 |
|  |  |  |  |  |  |  |
| **Women** |  |  |  |  |  |  |
| Paternal occ. class (4y) | 0.22 (0.02, 0.42) | 0.04 | -0.25 (-0.46, -0.04) | 0.02 | -0.04 (-0.27, 0.20) | 0.76 |
| Own education (26y) | 0.06 (-0.14, 0.27) | 0.55 | -0.16 (-0.38, 0.06) | 0.16 | -0.10 (-0.34, 0.15) | 0.45 |
| Occupational class (53y) | 0.08 (-0.14, 0.30) | 0.49 | -0.12 (-0.35, 0.10) | 0.28 | 0.02 (-0.24, 0.27) | 0.88 |
| Household income (60-64y) | -0.01 (-0.22, 0.19) | 0.93 | -0.07 (-0.28, 0.14) | 0.51 | -0.14 (-0.37, 0.10) | 0.26 |

|  | IGFBP3 | P | Morning cortisol | P | Diurnal cortisol (drop) | P |
| --- | --- | --- | --- | --- | --- | --- |
| **Men** |  |  | **Both sexes** |  | **Both sexes** |  |
| Paternal occ. class (4y) | 0.12 (-0.12, 0.37) | 0.31 | 0.09 (-0.08, 0.26) | 0.30 | 0.01 (-0.17, 0.14) | 0.86 |
| Own education (26y) | -0.13 (-0.37, 0.12) | 0.32 | -0.07 (-0.24, 0.10) | 0.40 | 0.06 (-0.09, 0.22) | 0.43 |
| Occupational class (53y) | 0.14 (-0.12, 0.40) | 0.29 | 0.15 (-0.02, 0.33) | 0.09 | -0.03 (-0.19, 0.13) | 0.70 |
| Household income (60-64y) | -0.25 (-0.49, -0.01) | 0.04 | -0.10 (-0.27, 0.06) | 0.22 | 0.14 (-0.01, 0.29) | 0.08 |
|  |  |  |  |  |  |  |
| **Women** |  |  |  |  |  |  |
| Paternal occ. class (4y) | -0.17 (-0.40, 0.07) | 0.17 |  |  |  |  |
| Own education (26y) | -0.17 (-0.42, 0.08) | 0.18 |  |  |  |  |
| Occupational class (53y) | -0.16 (-0.41, 0.09) | 0.21 |  |  |  |  |
| Household income (60-64y) | -0.23 (-0.46, 0.00) | 0.05 |  |  |  |  |

Notes: positive coefficients indicate that those of lower SEP tended to have higher hormone concentrations; #P-value for sex interaction term <0.05; occupational class was that of the highest in the household and derived using the Registrar General’s classification; N = 752 men, 745 women for total testosterone – those for other hormones differ slightly (see text).
